# Advances in the Functions and Molecular Mechanisms of SPL Transcription Factors in Plant Flower Development

**DOI:** 10.3390/plants15192975

**Published:** 2026-09-29

**Authors:** Qiufei Wu, Xiaohui Pan, Lixia Zhou

**Affiliations:** 1 State Key Laboratory of Tropical Crop Breeding, Chinese Academy of Tropical Agricultural Sciences, Sanya 572025, China; qfi_wu@catas.cn (Q.W.); 13976932556@163.com (X.P.); 2 Coconut Research Institute, Chinese Academy of Tropical Agricultural Sciences, Wenchang 571339, China

**Keywords:** SPL transcription factors, SBP-box domain, flower development, flowering regulation, molecular mechanism

## Abstract

SQUAMOSA PROMOTER BINDING PROTEIN-LIKE (SPL) transcription factors constitute a plant-specific family defined by a conserved SBP DNA-binding domain. They recognize *cis*-elements containing the core GTAC motif and cooperate with various co-regulators to govern downstream gene expression, thereby participating in leaf morphogenesis, phase transition, fruit development, anthocyanin accumulation and abiotic stress adaptation. Notably, the abbreviation SPL is also used for the unrelated SPOROCYTELESS/NOZZLE (SPL/NZZ) protein, which belongs to a separate family lacking the SBP-box domain; these two families should not be confused. Flower development, a central reproductive program in angiosperms, encompasses the acquisition of flowering competence, the vegetative-to-reproductive transition, floral meristem specification, organ patterning, and gametophyte formation. This review summarizes structural characteristics and classification of SPL proteins, distinguishes miR156/miR157-targeted paralogs from non-targeted ones, and synthesizes current understanding of their molecular functions across successive floral stages. Acting as signaling hubs, SPLs integrate photoperiod, gibberellin-DELLA, age-dependent miR156/miR157, temperature, sugar and cytokinin signals to fine-tune flowering time. They drive the reproductive transition via direct activation of *LFY*, *FUL* and *AP1*, and later remodel inflorescence architecture and floral organ size by balancing cell proliferation and expansion. In addition, they maintain fertility by modulating gametogenesis. Based largely on findings from *Arabidopsis thaliana*, we construct an updated regulatory framework for SPL-dependent floral development, compare conserved and lineage-divergent functions across herbaceous and woody species, identify unresolved questions, and discuss potential applications for crop improvement.

## 1. Introduction

Flowers represent one of the most complex organ systems in angiosperms and are indispensable for sexual reproduction, as they produce male and female gametophytes and provide the physical and nutritional environment for seed formation [1]. Most flowers consist of four whorls of floral organs arranged from the outermost to innermost whorl: sepals, petals, stamens, and carpels [2]. In recent decades, molecular genetics has greatly advanced our understanding of flower development, with the classic ABC model gradually expanded to the ABCDE model [3,4,5]. Flower development has thus become a highly active area of research in molecular biology and plant science [6].

SQUAMOSA PROMOTER BINDING PROTEIN-LIKE (SPL) transcription factors, also known as SBP-box transcription factors, are plant-specific and found from unicellular green algae to mosses, gymnosperms, and angiosperms. The first two SBP-family genes, *SBP1* and *SBP2* (SQUAMOSA PROMOTER BINDING PROTEIN), were isolated from snapdragon (*Antirrhinum majus*) inflorescences based on their capacity to bind the promoter of the MADS-box floral-regulator *SQUAMOSA* [7,8,9]. Subsequently, numerous SPL transcription factors have been identified across diverse plant species and shown to play critical roles in morphogenesis, flowering and floral organ development, fruit development and ripening, secondary metabolism, hormone signaling, and stress response.

SPL proteins are defined by the conserved SBP DNA-binding domain encoded by the SBP-box [9,10]. The number of family members varies substantially across taxa: 16 in *Arabidopsis thaliana* [10], 82 in alfalfa (*Medicago sativa* L.) [11], 19 in chickpea (*Cicer arietinum* L.) [12], and 24 in oil palm (*Elaeis guineensis*) [10]. In *Arabidopsis*, SPL protein sizes range from 131 (SPL3) to 927 (SPL12) amino acids [10]. Eleven *Arabidopsis* SPL transcripts are targeted by miR156 and are grouped into the SPL3 clade (*SPL3*/*4*/*5*) and the SPL9 clade (*SPL2*/*6*/*9*/*10*/*11*/*13*/*15*) [13]. The miR156 recognition site resides in the 3′ untranslated region (3′ UTR), mediating transcript cleavage and translational inhibition [14]. A subset of SPL transcripts also carries miR529/miR535 sites [15]. Beyond reproduction, SPLs participate in leaf development [16], phase transitions [17], fruit development [18,19,20], sporogenesis [21], gibberellin signaling [22], and copper stress responses [23].

## 2. Scope and Literature Search Strategy

### 2.1. Review Scope

This review synthesizes the conserved and lineage-specific functions of SPLs across the stages of flower development. In particular, we distinguish experimentally validated direct regulation from correlative evidence or evidence derived from heterologous overexpression. We also highlight major gaps and future directions. Unlike previous reviews that have focused mainly on the miR156-SPL cascade, this review gives balanced emphasis to model species, crops, and woody perennials [24].

### 2.2. Literature Search

A literature search was conducted in Web of Science, PubMed, Scopus, and the China National Knowledge Infrastructure (CNKI) using the following search terms: SPL transcription factor, SBP-box, miR156-SPL, flower development, floral transition, flowering time, floral organ, and fertility. Primary research articles were prioritized as sources of mechanistic evidence, whereas reviews were retained for background information. Studies published between 2018 and 2025 were given preference. Papers reporting only expression profiles without functional validation were excluded, except those included for evolutionary analyses [25].

## 3. Structural Characteristics and Functional Classification of SPL Transcription Factors

Transcription factors bind specific *cis*-elements to confer temporal, spatial, and dose-dependent regulation [26,27]. SPLs are restricted to green plants and characterized by an approximately 80-residue SBP domain [28], which is absent in prokaryotes, fungi, and animals.

**Figure 1:**
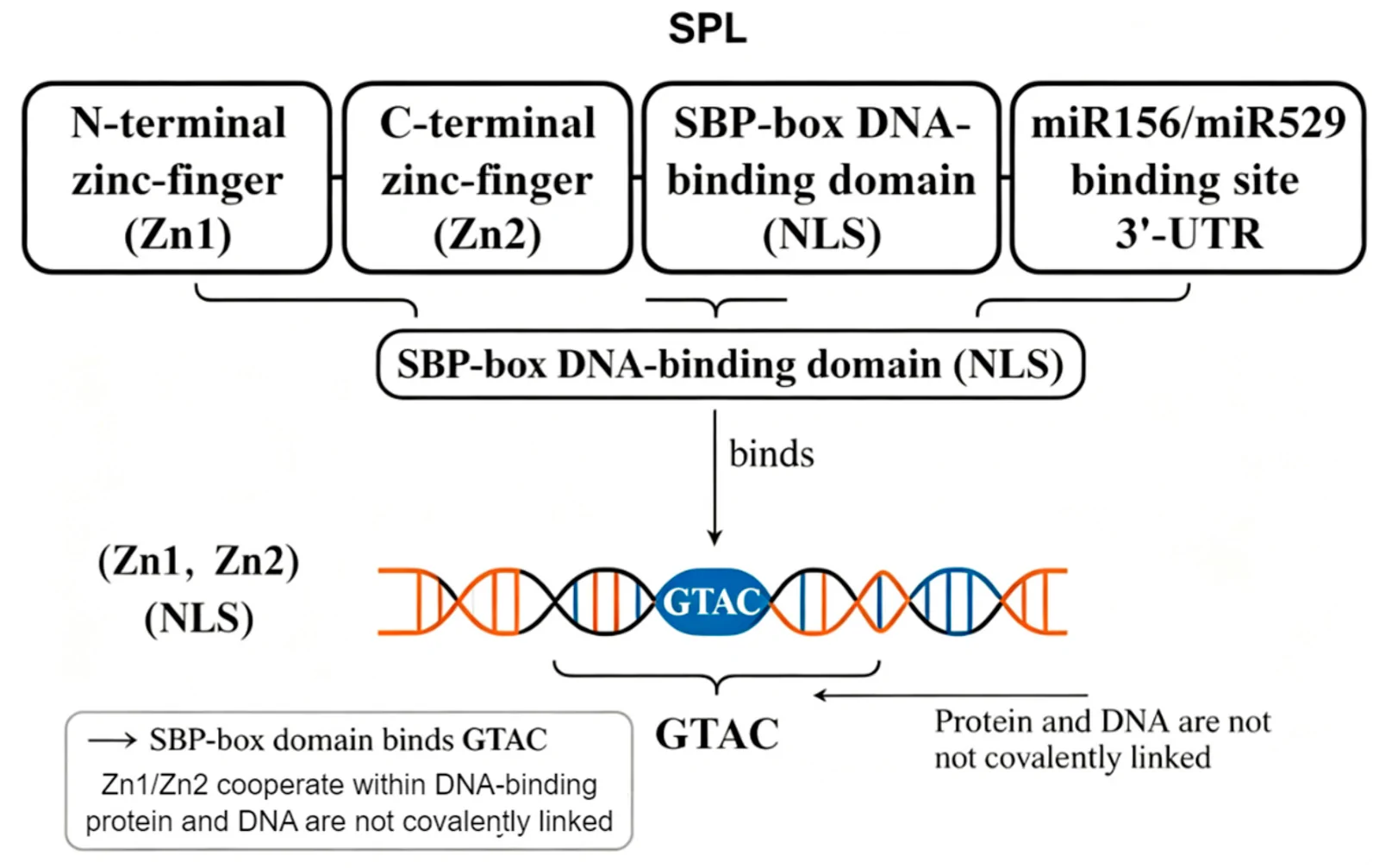
Schematic diagram of the conserved SBP-box domain of SPL transcription factors. Upper panel: domain organization of the SPL protein/gene. Lower panel: double-stranded DNA containing the GTAC core cis-element. A single arrow illustrates that the SBP-box domain mediates physical binding to the GTAC motif. Abbreviations: Zn1, N-terminal zinc finger; Zn2, C-terminal zinc finger; NLS, nuclear localization signal; 3′ UTR, 3′ untranslated region. This figure depicts protein–DNA binding interaction; no covalent linkage between SPL protein and DNA is implied.

The SBP domain adopts a dual zinc-finger architecture: eight cysteine/ histidine residues form two zinc fingers (Zn-1: Cys-Cys-Cys-His or Cys-Cys-Cys-Cys, Zn-2: Cys-Cys-His-Cys) [29,30]. Both modules are essential for DNA binding; loss of zinc abolishes activity [31]. This domain recognizes the *cis*-element TNCGTACAA, with GTAC as the invariant core [29]. A nuclear localization signal (NLS) partially overlaps Zn-2 [29]. Phosphorylation near the NLS modulates nuclear import and DNA binding [29]. Many SPLs carry miR156/miR157 sites, whereas a smaller subset carries miR529/535 sites [31,32,33]. The SBP-box coding region is split by a conserved intron [34] (Figure 1).

Based on miR156 targeting, *Arabidopsis* SPLs fall into two classes [35]. The first class comprises miR156-targeted SPLs (SPL3/4/5, SPL2/6/9/10/11/13/15), which are dynamically repressed in juveniles and de-repressed with age [34]. Among these, SPL3/4/5 are short proteins enriched in tissues undergoing floral transition [36], whereas SPL9 and SPL15 are longer proteins with broader roles [37]. The second class comprises non-miR156-targeted SPLs (SPL1/7/8/12/14), which escape miR156 repression. SPL14 negatively regulates floral transition [38], while *SPL8* plays critical roles in anther and ovule development [39].

Within the miR156-targeted group, two functionally distinct clades exist: the short SPL3/4/5 clade and the longer SPL9-clade (SPL2/9/10/11/13/15) [13]. SPL3/4/5 accumulate in shoot apical meristems upon floral induction and directly activate *LFY*, *FUL*, and *AP1* to drive floral meristem identity, and they play minor roles in vegetative phase change [40]. By contrast, members of the SPL9 clade are broadly expressed, drive the juvenile-to-adult transition, and mediate gibberellin- and cytokinin-dependent flowering [41]. Among non-targeted SPLs, *SPL8* functions primarily in male and female reproductive organ development, regulating microsporogenesis and megasporogenesis [13,39]. Others, such as *SPL7* (copper homeostasis) and *SPL14* (floral repression), have divergent roles [42,43].

Post-transcriptional repression by miR156/miR157 shapes three features of the SPL family: functional redundancy, tissue specificity, and developmental stage specificity. Single-gene mutants often show subtle phenotypes, whereas higher-order mutants display severe defects [8,41]. The spatiotemporal gradient of miRNA abundance restricts high SPL activity to specific cells and developmental windows. Non-targeted SPLs such as *SPL8* are decoupled from this age-dependent rheostat and are controlled predominantly at the transcriptional level [13].

## 4. SPL Regulatory Input–Output Networks During Plant Floral Development

Flowering is a multistep program integrating age, hormonal, and environmental cues [44]. At the molecular level, it proceeds through the following steps: (1) perception of inductive signals, (2) convergence upon meristem identity regulators, (3) activation of organ identity genes, and (4) downstream morphogenesis [45,46].

To clarify the logic of SPL-dependent regulation, we organize this section around a four-layer framework [47,48]: (1) upstream regulators (photoperiod, temperature, age, GA, sugar, cytokinin) that modulate SPL activity via miR156/157 and DELLA [37], (2) SPL integrators that process these inputs, (3) direct SPL targets validated by ChIP-seq or EMSA [49], and (4) secondary downstream outputs (floral transition, inflorescence architecture, organ morphogenesis, fertility) [50]. We distinguish direct regulation (supported by molecular evidence) from indirect effects (inferred from mutant phenotypes) [24].

### 4.1. Juvenile-to-Adult Phase Transition

The juvenile-to-adult phase transition is a vegetative phase change during which plants gain reproductive competence. The miR156-SPL module functions as a central molecular timer governing this process.

The miR156-miR172 cascade constitutes the core regulatory module of the age pathway, and its activity shows tissue- and cell-type specificity; these microRNAs also participate in multiple biological processes beyond age-dependent phase transition. High miR156 levels in juveniles maintains low SPL activity, whereas declining miR156 levels with age de-repress SPLs to promote adult traits [51]. Members of the SPL9 clade are major drivers of this transition [41]. SPL9 and SPL10 directly activate the transcription of MIR172b [44].

SPLs also regulate cauline leaf identity. miR156-targeted SPLs (SPL2/9/10/11/13/15) redundantly regulate cauline leaf morphology. *SOC1* and *FUL* directly bind and activate *SPL9* and *SPL15*, thereby linking age signaling to leaf identity. SPL9 directly targets BRI1, forming the SOC1/FUL-SPL-BRI1 module [41].

Sugars act as endogenous age signals. Sucrose, glucose, and maltose repress the accumulation of primary transcripts for most *MIR156* genes [52]. Cytokinin and gibberellin interact during the phase transition; cytokinin responses require GA biosynthesis and DELLA function [53].

Recent studies have revealed functional divergence between miR156 and miR157. In the shoot apical meristem, miR156 declines more rapidly with age than miR157. CRISPR-generated *mir157c* mutants flower early under short days, whereas mir156ac mutants do not. The *MIR157C*-*SPL15* module is conserved in *Arabis alpina* [54].

### 4.2. Vegetative-to-Reproductive Transition

**Figure 2:**
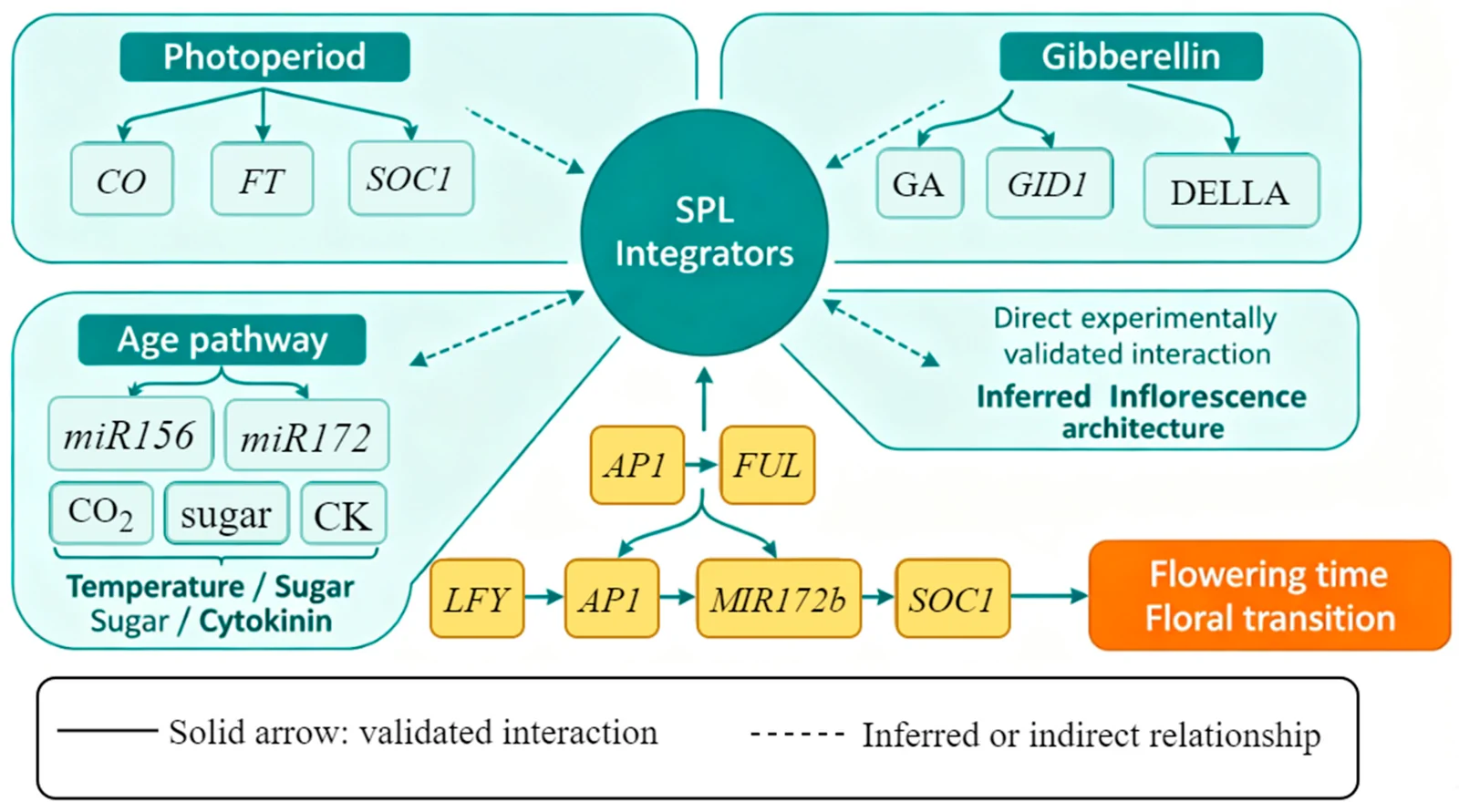
SPL hub integrating photoperiod, gibberellin, age, temperature, and sugar signals to regulate flowering time in *Arabidopsis.* GA: gibberellin; GID1: GA receptor; DELLA: DELLA repressor; miR: microRNA; SOC1: SUPPRESSOR OF CONSTANS 1; FT: FLOWERING LOCUS T; CO: CONSTANS. Solid arrows indicate activation; blunt bars indicate repression.

After acquiring competence, the shoot apical meristem ceases vegetative growth and initiates reproductive development. Five major pathways—photoperiod, vernalization, gibberellin, autonomous, and age-dependent miR156-miR172—converge on SPL activity (Figure 2) [55].

#### 4.2.1. Photoperiod Pathway

In *Arabidopsis*, photoperiod signaling activates *GI* and *CO*, which drive *FT* expression in leaves. PHYA and PHYB exert antagonistic effects [56]. FT protein moves to the shoot apical meristem (SAM), where it interacts with FD to activate *SOC1* [57,58]. ABA acts synergistically with *GI* to enhance the association of *CO* with the *FT* promoter [59]. *VQ18* and *VQ26* stabilize CO and relieve *TOE1*/*TOE2*-mediated repression [60].

The transcript levels of *SPL3*/*4*/*5* are low under short days and elevated under long days. *SOC1* directly binds the promoters of these genes to activate transcription [22]. Under short days, GA promotes flowering via this SOC1-SPL module, but this effect is weak under long days [22]. Photoperiod-dependent induction also requires the homeodomain factors PNY and PNF [61]. In snapdragon, silencing the *SPL3*/*4*/*5* ortholog *AmSBP1* delays flowering [62].

#### 4.2.2. Gibberellin-DELLA-SPL Signaling

GA acts as a ligand and binds to its receptor GID1. The resulting GA-GID1 complex physically interacts with DELLA repressors, targeting DELLA for ubiquitin-dependent degradation via the SCF^GID2^-proteasome system, this degradation removes DELLA-imposed repression and thereby releases SPL transcription factors [48]. Under short days, GA-triggered DELLA degradation relieves repression of *SPL9*, which up-regulates *SOC1* and miR172b to accelerate flowering [22,48]. IDD14/15/16 interact with DELLA and constitute additional GA signaling components [63]. In wheat, DELLA proteins interact with SPL homologs to regulate heading date [64].

Elevated CO_2_ reduces miR156/miR157 via sugar signaling and elevates miR172 and SPL expression [65]. Low temperature (16 °C) enhances miR156 accumulation and represses SPLs [66]. Root-derived cytokinin promotes flowering via the two-component system, requiring *SPL15* and *miR172*, independently of photoperiod [51]. In rose, *SPL3*/*4*/*5* participate in first flowering time [67].

SPLs show functional divergence during this transition. The non-targeted *SPL14* acts as a negative regulator [50]. DELLA proteins physically interact with SPLs and repress SPL activity, delaying flowering [48,64].

### 4.3. Floral Meristem Initiation and Identity

After the vegetative-to-reproductive transition, the inflorescence meristem produces floral meristems. At this stage, SPLs directly activate key floral meristem identity genes.

As miR156 declines, de-repressed SPLs promote floral meristem identity by activating *LFY*, *FUL*, and *AP1* [50,53]. *LFY* is a direct target of *SPL3*. Overexpression of *SPL3* increases *LFY* expression but cannot fully rescue the *lfy* mutant phenotype, suggesting the involvement of parallel pathways [68]. *SPL9* and *SPL10* directly activate *MIR172b* transcription [44].

In maize, *Glossy15* (*Gl15*) acts downstream of the miR156-SPL-miR172 cascade. *Gl15* delays flowering by repressing GA biosynthesis and floral organ genes. The elite early-flowering haplotype Hap1 has been under positive selection [37,69].

**Figure 3:**
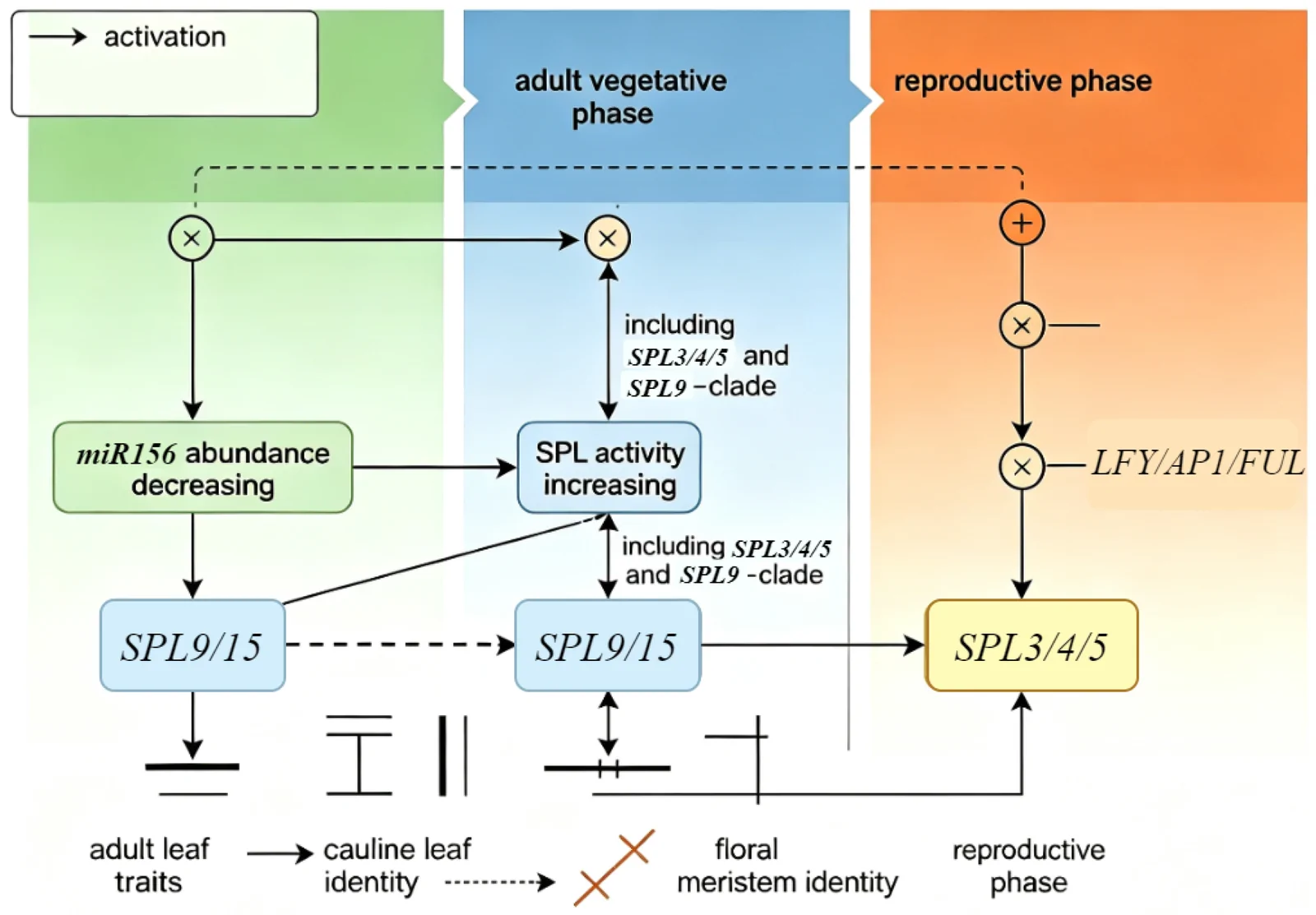
Model for miR156-SPL-mediated phase transitions in *Arabidopsis*. Abbreviations: miR: microRNA; SAM: shoot apical meristem; LFY: LEAFY; AP1: APETALA1; FUL: FRUITFULL. Solid arrows indicate validated interactions; dashed arrows indicate inferred relationships; blunt bars indicate repression.

In woody perennials, heterologous overexpression of *PaSPL8a* from plane tree accelerates flowering [70]. However, these observations provide only preliminary evidence; loss-of-function evidence in native species is still required (Figure 3).

### 4.4. Floral Organ Specification and Patterning

Once floral meristem identity is established, downstream regulatory cascades drive organ patterning, thereby shaping inflorescence architecture and pedicel morphology and determining organ size. Most of these outputs represent secondary effects of SPL-regulated target genes.

Overexpression of *SPL3* accelerates flowering and produces abnormal floral and inflorescence phenotypes. Rice *OsSPL13* regulates inflorescence and flower development. Snapdragon *AmSBP1* contributes to floral primordium initiation [71]. In the orchid *Cymbidium sinense*, the *CsSEP4*-*CsSPL18* regulatory module contributes to gynostemium morphological variation [72]. Heterologous expression of loquat *EjSPL3*/*4*/*5*/*9* and plane tree *PaSPL8a* promotes flowering in *Arabidopsis* [73,74].

The final floral organ size is determined by cell proliferation and expansion. miR156-repressed *SPL2* modulates petal, sepal, and silique elongation. *SPL2* directly binds the 5′ UTR of *AS2* to regulate its transcription [73]. *PNY*/*PNF* activate *SPL3*/*4*/*5* and repress *miR156* [75]. *KNAT1* and *KNAT2* contribute to organ initiation, and *AS2* is repressed by *KNAT1* [62]. *LFY* also binds to *AS2* regulatory regions to prevent excessive growth [76].

**Figure 4:**
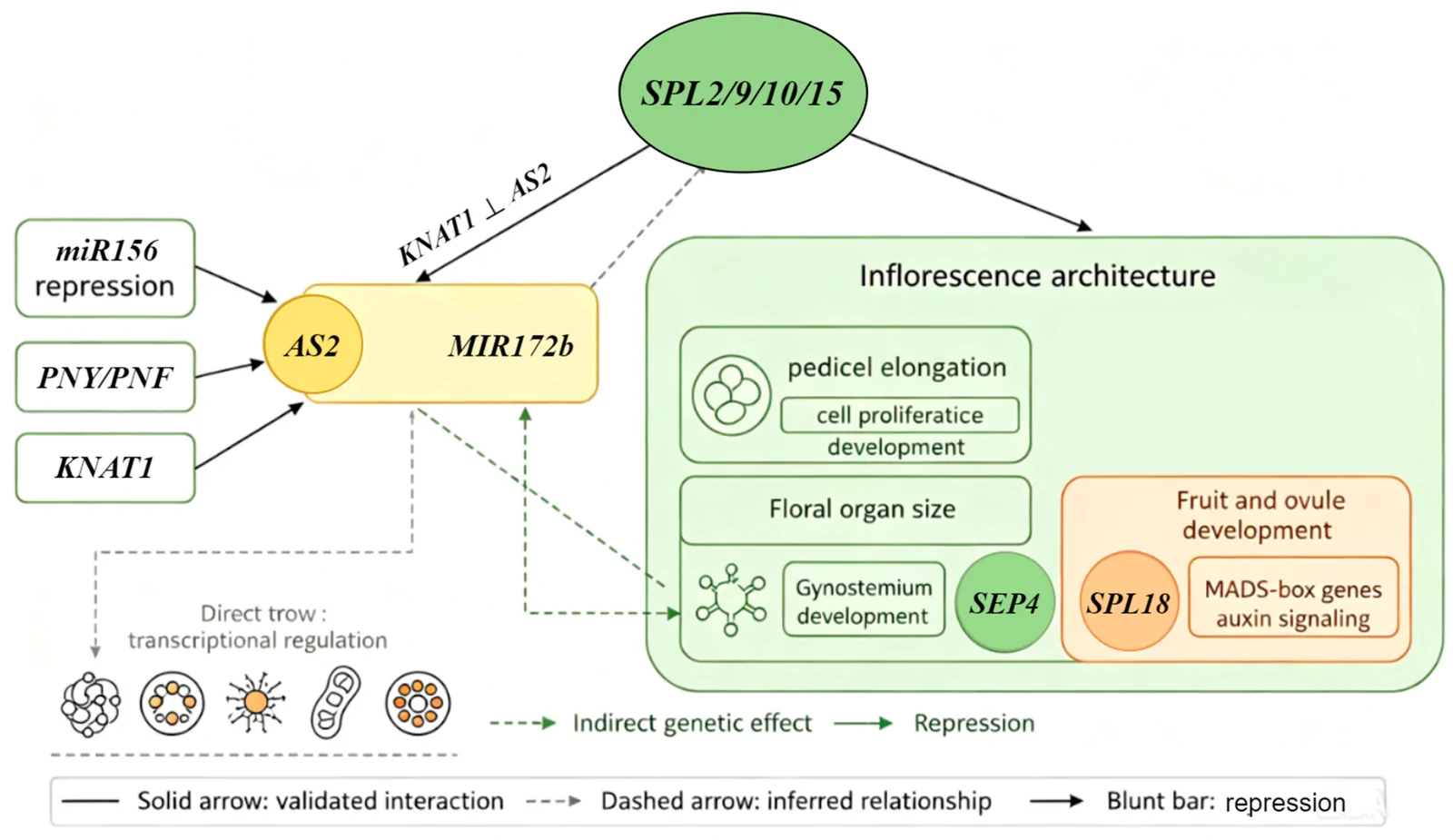
Regulatory network of SPL transcription factors in inflorescence architecture, pedicel elongation, and floral organ growth in *Arabidopsis thaliana*. Abbreviations: miR: microRNA; PNY: PENNYWISE; PNF: POUND-FOOLISH; AS2: ASYMMETRIC LEAVES 2; KNAT: KNOTTED-like; SEP4: SEPALLATA4. Solid arrows indicate validated interactions; dashed arrows indicate inferred relationships; blunt bars indicate repression.

Manipulation of the miR156-SPL module alters cell proliferation and expansion: miR156 overexpression increases cell size while reducing cell number; SPL overexpression increases cell number and reduces cell size. The miR157-SPL cascade modulates organ size and ovule development by tuning MADS-box and auxin signaling [77]. Overexpression of cotton *GhmiR157* reduces petal area, pistil length, and ovule number [78]. *35S:MIR156b* transgenic *Arabidopsis* plants develop shorter pistils and fewer ovules [79]. By contrast, overexpression of tomato *LeMIR156b* does not reduce floral organ size but influences fruit development, illustrating lineage-specific divergence [80]. In tomato, de-repressed SPL activates MADS-box genes and enhances auxin signaling to support ovule formation [81] (Figure 4).

### 4.5. Male and Female Reproductive Development

Following floral organ patterning, stamens and carpels differentiate further. Notably, among non-miR156-targeted SPLs, *SPL8* plays a central and largely age-independent role in reproductive organ differentiation, with partial functional redundancy provided by miR156-targeted SPLs [79].

#### 4.5.1. Distinct Roles of SPL8 in Male vs Female Development

Male functions. *spl8* loss-of-function mutants exhibit defects in pre-meiotic microsporangium differentiation, tapetal specification, and pollen production, resulting in partial male sterility [14,40]. In addition, both loss- and gain-of-function *SPL8* alleles impair filament elongation and anther dehiscence, indicating that proper anther function depends on finely tuned SPL8 expression levels [82]. Importantly, no published ChIP-seq or EMSA has confirmed direct binding of *SPL8* to anther gene in *Arabidopsis*; all conclusions derive from genetic analyses.

Female functions. *spl8* mutants show disrupted differentiation of megaspore mother cells and reduced ovule viability [39]. Similarly, these conclusions are supported solely by genetic evidence, and their direct targets remain uncharacterized [79].

#### 4.5.2. Genetic Interactions with miR156-Targeted SPLs

SPL8 acts in parallel and partially redundantly with miR156-targeted SPLs (SPL2/9/15). Introducing miR156 overexpression into the *spl8* mutant background results in near-complete sterility, revealing genetic redundancy [79]. However, existing data do not support a physical protein–protein interaction between SPL8 and these SPLs; the redundancy appears to operate at the level of parallel transcriptional inputs. *SPL8* also integrates GA signaling via DELLA repressors [14].

### 4.6. Gametogenesis and Fertility

**Figure 5:**
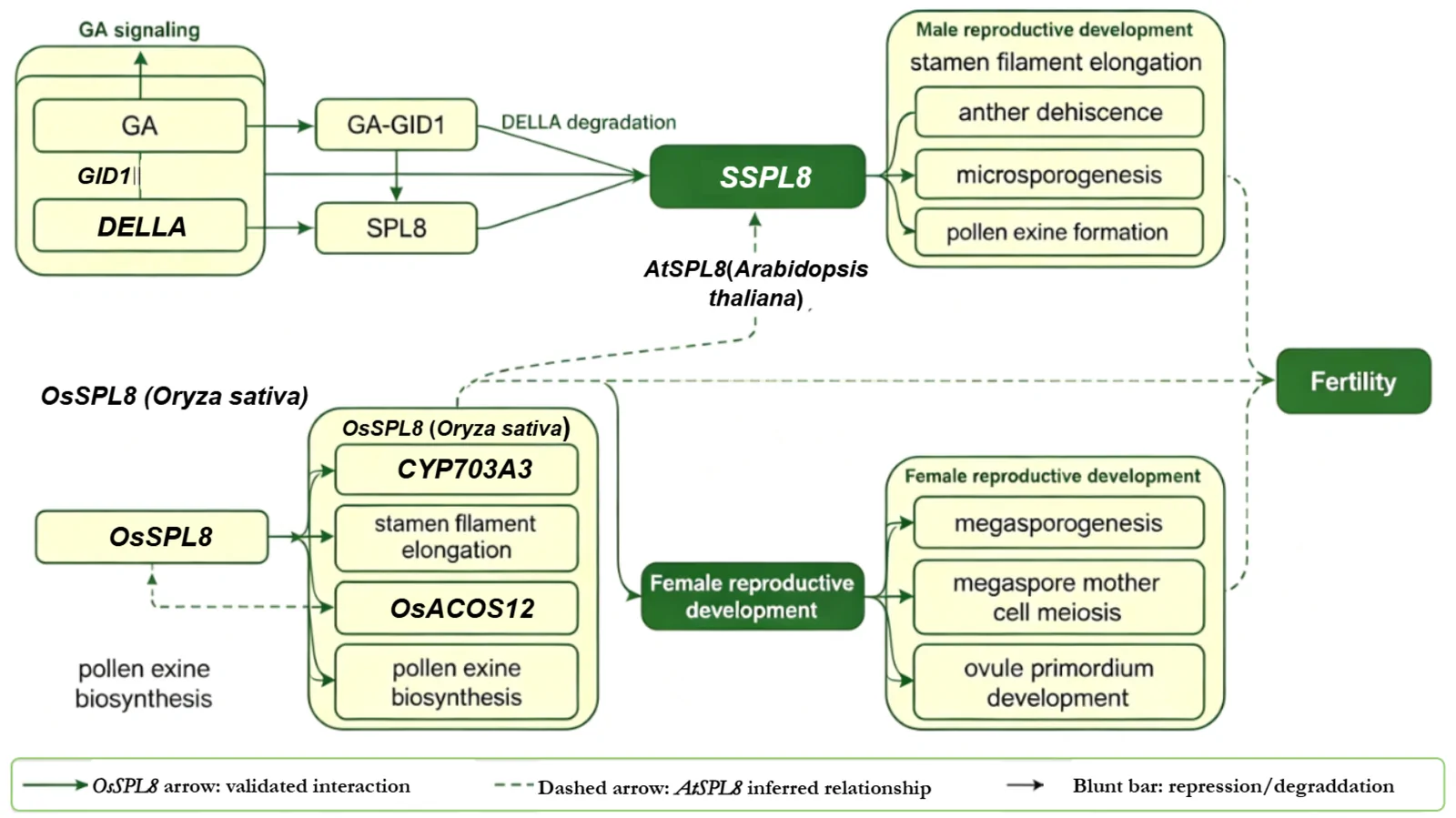
*SPL8* pathways in reproductive development of *Arabidopsis* and rice. GA: gibberellin; GID1: GA receptor; Solid arrows indicate validated interactions (*OsSPL8* direct targets); dashed arrows indicate inferred relationships (*AtSPL8* genetic evidence); blunt bars indicate repression.

In *Arabidopsis spl8* mutants, meiotic initiation of megaspore mother cell is frequently blocked, and pollen-sac wall formation is defective [39]. These observations are grounded in genetic evidence; the direct DNA-binding targets governing meiosis remain to be validated. By contrast, rice *OsSPL8* has been functionally characterized, with validated direct targets in stress responses (e.g., *OsGS1;1*, *OsNAC17*, and *OsOMTN6*) [82]. Loss of *OsSPL8* function has been associated with reproductive defects, and OsSPL8 may regulate genes involved in pollen wall formation; however, direct targets such as *CYP703A3* and *OsACOS12* remain to be experimentally validated [83]. Unlike *Arabidopsis* SPL8, whose functions in both male and female reproductive development are well documented [14], rice *OsSPL8* has been primarily characterized in vegetative development and stress responses; its role in female gametogenesis remains poorly characterized, and direct comparisons of functional divergence between these two orthologs warrant further investigation (Figure 5).

## 5. Discussion

The hierarchical SPL network underscores their role as signaling hubs. Upstream signals converge via miR156/157 and GA-DELLA interactions. SPLs directly regulate target genes that coordinate floral development. This multilayered system enables flexible control of flowering time and organogenesis.

### 5.1. Core Conserved Functions of SPL Proteins

Among angiosperms, the miR156-targeted subset of SPL genes is broadly conserved, and miR156-dependent de-repression of SPL transcripts during developmental aging represents an ancient regulatory module of the age pathway [16,84]. The best-documented conserved function of SPL proteins is promoting the juvenile-to-adult vegetative phase transition and subsequent floral induction. In *Arabidopsis thaliana*, SPL3/4/5 directly activate the floral meristem identity genes LFY, FUL, and AP1 to drive the reproductive transition [68], and this regulatory logic appears to be at least partially conserved in rice, maize, and woody perennials [56]. Beyond the phase transition, a second conserved function is the integration of multiple flowering signals. SPLs perceive and integrate inputs from photoperiod, GA-DELLA signaling, age-dependent miR156/miR157, temperature, sugar, and cytokinin, positioning them as integrative nodes that translate diverse physiological and environmental cues into developmental outcomes [56].

### 5.2. Species-Specific and Lineage-Divergent Functions

Despite these core conserved roles, however, there is considerable lineage-specific divergence. For example, in tomato, the miR156-SPL cascade primarily modulates fruit development rather than floral organ size [85], whereas in cotton, miR157-SPL signaling primarily regulates ovule and floral organ growth [81]. In cereals, SPL homologs act as key determinants of heading date and panicle architecture, traits for which there are no direct equivalents in *Arabidopsis* [86]. Recent DAP-seq analyses have provided molecular insights into the basis of this divergence. Comparative analysis of SPL homologs in *Arabidopsis*, maize, and wheat revealed that although some target genes are conserved, the DNA motifs recognized by SPLs can be species-specific. Notably, wheat SPLs preferentially bind longer palindromic motifs distinct from the GTAC core motif that is preferred by *Arabidopsis* SPLs, a divergence that may arise from enhanced protein–protein interactions stabilizing dimeric binding [87]. Collectively, these findings suggest that SPL family members have diversified in part through evolution of DNA-binding properties, acquiring species-specific regulatory programs that reflect lineage-specific developmental requirements.

### 5.3. Determinants of Functional Specificity Among Closely Related SPLs

Functional specificity among closely related SPL paralogs can arise from multiple mechanisms, including gain or loss of miRNA target sites, divergence in tissue-specific promoter activity, differences in protein–protein interaction partners, altered DNA-binding preferences, and subfunctionalization following gene duplication [88,89]. For instance, a comparative analysis of the closely related paralogs *AtSPL9* and *AtSPL15* has uncovered divergence in DNA-binding specificity and target gene regulation, particularly in the auxin and abscisic acid pathways [87]. Despite their high sequence similarity, these two SPLs recognize distinct DNA motifs and regulate partially non-overlapping sets of target genes, indicating that even within the same species and clade, SPL members have acquired functional specialization through subtle differences in their DNA-binding properties [87]. Similarly, the SPL3/4/5 clade and the SPL9 clade exhibit marked functional divergence: the former are shorter proteins enriched in tissues undergoing floral transition and primarily promote floral meristem identity, whereas the latter are longer, broadly expressed proteins that are major drivers of the juvenile-to-adult vegetative phase transition [37].

### 5.4. Directly Demonstrated SPL–Target Relationships

Distinguishing between experimentally validated direct regulation and inferred indirect associations is critical. A subset of regulatory relationships is supported by multiple independent high-confidence experimental approaches, including genetic loss-of-function mutant analyses, electrophoretic mobility shift assays (EMSA), chromatin immunoprecipitation followed by sequencing (ChIP-seq), and complementation assays. The most firmly established direct SPL–target relationships include: (1) *LFY*, *AP1*, and *FUL* as direct targets of *SPL3*/*4*/*5*, validated by chromatin immunoprecipitation (ChIP) and EMSA in *Arabidopsis*) [68]; (2) *MIR172b* as a direct target of *SPL9* and *SPL10* [44]; (3) *AS2* as a direct target of *SPL2* via binding to the 5′-UTR of AS2 [73]; and (4) well-documented direct targets of OsSPL8 in stress responses (e.g., *OsGS1;1*, *OsNAC17*, and *OsOMTN6*) as validated by EMSA and ChIP-PCR [82]. Loss of OsSPL8 function has been associated with reproductive defects, but whether OsSPL8 directly regulates genes involved in pollen wall formation, such as *CYP703A3* and *OsACOS12*, remains to be experimentally validated [82]. In contrast, the regulatory roles of *AtSPL8* in anther dehiscence, stamen filament elongation, and megasporogenesis are supported solely by genetic analyses of mutants, no published ChIP-seq or EMSA has confirmed direct physical binding of *AtSPL8* to the promoters of structural genes involved in anther development in *Arabidopsis* [39]. Thus, while the genetic requirement for *AtSPL8* in reproductive development is clear, its direct molecular targets remain biochemically uncharacterized.

### 5.5. Uncertain and Controversial Mechanisms

Several proposed SPL-mediated mechanisms remain incompletely resolved and require further validation. First, although DELLA proteins physically interact with SPLs and repress their transcriptional activity, it remains unclear whether they directly block SPL DNA-binding, recruit co-repressors, or affect SPL protein stability [48]. Studies in wheat have shown that MADS-box SQUAMOSA family proteins VRN1 and FUL2 can bind DELLA proteins and competitively alleviate DELLA-imposed repression of SPLs; however, whether this mechanism is conserved across species requires further investigation [64]. Second, the functional distinction between *miR156* and *miR157* remains unclear. Recent findings indicate that RISC-loaded *miR156* declines more rapidly with plant age than *miR157*, and *miR157* maintains relatively high abundance during floral transition and inflorescence development [54]. The MIR157C-SPL15 module appears to have specialized roles in short-day-induced floral transition that is conserved in *Arabis alpina*; yet the extent to which *miR156* and *miR157* have genuinely distinct targets as opposed to overlapping but temporally separated functions remains an open question [54,90]. Third, the emerging roles of SPLs in stress responses, including *AtSPL14*-mediated tolerance to fumonisin B1, *AtSPL9*-facilitated reactive oxygen species (ROS)–salicylic acid (SA) signaling in immunity, and OsSPL4-dependent upregulation of defense-associated genes for blast resistance, are supported primarily by genetic evidence [24,91]; for many of these functions, whether SPL involvement is direct or indirect remains unclear, and downstream target genes remain largely uncharacterized. Fourth, observations from heterologous overexpression experiments in *Arabidopsis* have provided preliminary evidence for SPL functions in litchi, loquat, plane tree, and coconut [70,73,92,93]. However, phenotypes obtained via heterologous overexpression cannot substitute for loss-of-function genetic evidence obtained in native woody species. Therefore, whether the SPL-dependent flowering regulatory logic observed in herbaceous models applies to perennial woody taxa requires direct genetic validation in those species [94,95].

### 5.6. Convergence of Environmental and Hormonal Pathways on SPL Activity

SPL transcription factors function as points of convergence for multiple upstream signals. Five major genetically defined flowering-induction pathways converge on SPL activity. These include the photoperiod, vernalization, gibberellin, and autonomous pathways, as well as the age-dependent miR156-miR172 cascade. In the miR156/miR157-mediated age pathway, *miR156* abundance decreases with plant age, progressively de-repressing SPL transcripts and forming a core developmental timing mechanism that ensures flowering occurs at an appropriate ontogenetic stage [48,49]. In GA-DELLA signaling, GA binds to the GID1 receptor, and the GA-GID1 complex targets DELLA proteins for ubiquitin-mediated proteasomal degradation, thereby releasing SPLs from DELLA-mediated inhibition [56]; under short-day conditions, this GA-triggered degradation plays a dominant flowering-promoting role by relieving repression of *SPL9*. In the photoperiod pathway, *SOC1* directly binds the promoters of *SPL3*/*4*/*5* to activate their transcription, forming the SOC1-SPL regulatory module [22]; photoperiod-dependent induction additionally requires the homeodomain transcription factors *PNY* and *PNF*. Sugar signaling also contributes: sucrose, glucose, and maltose repress the accumulation of primary transcripts for most MIR156 family members, thereby providing a metabolic signal linking carbohydrate status to developmental timing [52]. Finally, root-derived cytokinin promotes flowering via the two-component signaling system, requires age-pathway components *SPL15* and *miR172*, and acts independently of the photoperiod pathway. This convergence suggests that SPL proteins are not simply downstream effectors of individual pathways but rather integrative nodes that process developmental decisions based on multiple inputs [43]. The molecular basis of this integration, whether additive, synergistic, or gated, remains an important unresolved question.

### 5.7. Major Unresolved Questions

Several critical questions remain for future investigation. (1) How does the miR157-SPL regulatory module collaborate with other uncharacterized cofactors to drive floral organ development? The functional distinction between *miR156* and *miR157* targets is only beginning to be understood [55]. (2) What are the molecular mechanisms balancing outputs from the miR156-SPL flowering-time cascade against other independent flowering-induction pathways, and how are conflicting signals integrated at the SPL level [49]? (3) What are the direct DNA-binding targets of *AtSPL8* during anther and ovule development? Despite clear genetic evidence for *AtSPL8* function in reproductive development, its biochemical targets remain uncharacterized. (4) To what extent do SPL DNA-binding specificities differ between species, and how do these differences contribute to functional divergence? The recent finding that wheat SPLs bind palindromic motifs distinct from the GTAC core motif recognized by *Arabidopsis* SPLs raises important questions about the evolution of SPL target repertoires [87]. (5) How can knowledge of SPL regulatory networks be translated into crop breeding to optimize flowering time, inflorescence architecture, and yield-associated fertility traits? The rice Ideal Plant Architecture 1 (*IPA1*/*OsSPL14*) provides a proof of concept that SPL manipulation can simultaneously improve yield and stress tolerance, but applicability to other crops remains to be demonstrated [18]. (6) What is the mechanistic basis of SPL-mediated stress responses, and how do developmental and stress-related SPL functions intersect? The expanding repertoire of SPL functions in abiotic and biotic stress responses raises the possibility that SPLs integrate developmental decisions with environmental adaptation [96,97].

### 5.8. Exploiting SPL Pathways for Crop Improvement

SPL transcription factors are promising targets for crop improvement through gene editing and molecular breeding, and several lines of evidence support this potential [98]. The case of rice *OsSPL14* (*IPA1*) is particularly instructive: a single transcription factor promotes both yield and disease resistance in rice by simultaneously optimizing plant architecture (reducing tiller number while increasing grain yield per panicle) and activating *WRKY45* to enhance blast disease resistance [17,99]. This demonstrates that SPL manipulation can achieve trait integration, simultaneously improving multiple agronomic traits. The maize Glossy15 (Gl15) provides another example: Gl15 functions downstream of the miR156-SPL-miR172 cascade and delays flowering by repressing gibberellin biosynthesis and genes involved in floral organ development, and the elite early-flowering haplotype Hap1 has been under positive selection in modern Chinese and US maize breeding programs [69]. In tomato, the miR156-SPL module plays a primary role in fruit development rather than in floral organ size regulation. Overexpression of miR156 results in defects in floral meristem determinacy and increased fruit locule number, indicating that this pathway has been co-opted for fleshy fruit development [85]. Furthermore, specific miR156-targeted SPL/SBP genes, such as *SlSBP15* and *SlSPL-CNR*, have been shown to regulate fruit patterning and ripening, respectively [51,85]. These findings suggest that the SPL regulatory network can be tailored to species-specific breeding targets, providing a molecular basis for fine-tuning fruit traits that were important during tomato domestication [94]. Furthermore, CRISPR/Cas9-mediated knockout of SPL genes has already been demonstrated in various species [81]; in *Nicotiana benthamiana*, knockout of the *SPL13* clade consistently delays flowering initiation by approximately five days, demonstrating the feasibility of SPL manipulation in non-model species [100]. The path forward for crop improvement will require precise editing of SPL genes to modulate flowering time without deleterious pleiotropic effects, engineering of SPL–target gene networks to improve yield-associated traits, and harnessing SPL-mediated stress tolerance for climate-resilient agriculture [98,101]. Advances in gene-editing tools, single-cell transcriptomics, spatial transcriptomics, and high-throughput protein–DNA interaction assays will continue to enhance both mechanistic understanding and translational applications of SPL transcription factor functions throughout plant development.

### 5.9. Concluding Perspectives

Over the past two decades, studies on SPL transcription factors have progressed from gene identification to network-level understanding, revealing several key conceptual advances. First, the miR156-SPL-miR172 cascade constitutes an ancient ontogenetic timing module that coordinates vegetative phase change and floral competence across angiosperms [98,102]. Second, SPLs act as integrative hubs where multiple flowering pathways (photoperiod, gibberellin, age, sugar, and cytokinin) converge, enabling plants to integrate developmental decisions from diverse internal and external inputs [103]. Third, DNA-binding specificity has diversified during evolution, with lineage-specific motifs (e.g., palindromic sites in wheat versus GTAC core in *Arabidopsis*) contributing to species-specific regulatory programs [87]. Fourth, the functional division between miR156-targeted SPLs (phase transition and floral meristem identity) and non-targeted SPLs such as SPL8 (reproductive organ differentiation) reveals a hierarchical buffering system that safeguards fertility [79,104].

Despite these advances, several gaps persist. The biochemical targets of AtSPL8 remain uncharacterized, and the mechanistic basis of DELLA-SPL interaction remains debated. The functional distinction between miR156 and miR157 requires further investigation, as does the extent to which SPL-mediated stress responses intersect with developmental regulation [54,80]. Moreover, the translational potential of SPL manipulation in woody perennials and major crops other than rice has yet to be realized. Addressing these gaps will require integration of biochemical, genetic, and multi-omics approaches across a broader phylogenetic spectrum. Advances in gene editing, single-cell and spatial transcriptomics, and high-throughput protein–DNA interaction assays will continue to enhance both mechanistic understanding and translational applications of SPL functions in plant development [16,105].

## Data Availability

No new data were created or analyzed in this study. Data sharing is not applicable to this article.

## Abbreviations

SPL	SQUAMOSA PROMOTER BINDING PROTEIN-LIKE
SPL/NZZ	SPOROCYTELESS/NOZZLE
SBP	squamosa promoter-binding protein
Zn-1	Cys-Cys-Cys-His or Cys-Cys-Cys-Cys
Zn-2	Cys-Cys-His-Cys
miR	microRNA
PNY	PENNYWISE
PNF	POUND-FOOLISH
AS2	ASYMMETRIC LEAVES 2
KNAT	KNOTTED-like
SEP4	SEPALLATA4
GA	gibberellin
GID1	GA receptor
EMSA	electrophoretic mobility shift assay
ChIP-PCR	chromatin immunoprecipitation polymerase chain reaction
